# Temporal changes in cardiovascular disease and infections in dialysis across a 22-year period: a nationwide study

**DOI:** 10.1186/s12882-021-02537-1

**Published:** 2021-10-15

**Authors:** Kamal Preet Kaur, Mavish Safdar Chaudry, Emil Loldrup Fosbøl, Lauge Østergaard, Christian Torp-Pedersen, Niels Eske Bruun

**Affiliations:** 1 Department of Cardiology, Zealand University Hospital, Sygehusvej 10, 4000 Roskilde, Denmark; 2 Department of Cardiology, Herlev-Gentofte Hospital University of Copenhagen, Copenhagen, Denmark; 3 The Heart Center, University Hospital of Copenhagen, Rigshospitalet, Copenhagen, Denmark; 4 Department of Cardiology and Clinical Epidemiology, Aalborg University Hospital, Aalborg, Denmark; 5 Department of Health Science and Technology, Aalborg University, Aalborg, Denmark; 6 Clinical Institute, University of Copenhagen, Copenhagen, Denmark; 7 Clinical Institute, Aalborg University, Aalborg, Denmark

**Keywords:** Hemodialysis, Peritoneal dialysis, End stage kidney disease, Cardiovascular disease, Infective endocarditis, Pneumonia, sepsis

## Abstract

**Background:**

Cardiovascular diseases (CVD) and infections are recognized as serious complications in patients with end stage kidney disease. However, little is known about the change over time in incidence of these complications. This study aimed to investigate temporal changes in CVD and infective diseases across more than two decades in chronic dialysis patients.

**Methods:**

All patients that initiated peritoneal dialysis (PD) or hemodialysis (HD) between 1996 and 2017 were identified and followed until outcome (CVD, pneumonia, infective endocarditis (IE) or sepsis), recovery of kidney function, end of dialysis treatment, death or end of study (December 31st, 2017). The calendar time was divided into 5 periods with period 1 (1996–2000) being the reference period. Adjusted rate ratios were assessed using Poisson regression.

**Results:**

In 4285 patients with PD (63.7% males) the median age increased across the calendar periods from 65 [57–73] in 1996–2000 to 69 [55–76] in 2014–2017, (*p* <  0.0001). In 9952 patients with HD (69.2% males), the overall median age was 71 [61–78] without any changes over time.

Among PD, an overall non-significant decreasing trend in rate ratios (RR) of CVD was found, (*p* = 0,071). RR of pneumonia increased significantly throughout the calendar with an almost two-fold increase of the RR in 2014–2017 (RR 1.71; 95% CI 1.46–2.0), (*p* <  0.001), as compared to the reference period. The RR of IE decreased significantly until 2009 (RR 0.43; 95% CI 0.21–0.87), followed by a return to the reference level in 2010–2013 (RR 0.87; 95% CI 0.47–1.60 and 2014–2017 (RR 1.1; 95% CI 0.59–2.04). A highly significant (*p* <  0.001) increase in sepsis was revealed across the calendar periods with an almost 5-fold increase in 2014–2017 (RR 4.69 95% CI 3.69–5.96).

In HD, the RR of CVD decreased significantly (*p* <  0.001) from 2006 to 2017 (RR 0.85; 95% CI 0.79–0.92). Compared to the reference period, the RR for pneumonia was high during all calendar periods (*p* <  0.05). The RR of IE was initially unchanged (*p* = 0.4) but increased in 2010–2013 (RR 2.02; 95% CI 1.43–2.85) and 2014–2017 (RR 3.39; 95% CI 2.42–4.75). No significant changes in sepsis were seen.

**Conclusion:**

Across the two last decades the RR of CVD has shown a decreasing trend in HD and PD patients, while RR of pneumonia increased significantly, both in PD and in HD. Temporal trends of IE in HD, and particularly of sepsis in PD were upwards across the last decades.

**Supplementary Information:**

The online version contains supplementary material available at 10.1186/s12882-021-02537-1.

## Introduction

Cardiovascular disease (CVD) and infections remain the two main leading causes of mortality in hemodialysis patients [1–3]. Cardiovascular causes - including arrhythmias, sudden cardiac death, congestive heart failure, myocardial infarction and atherosclerotic heart disease - were responsible for 48% of deaths among dialysis patients according to the US 2018 Annual Data Report [4]. Annual death rates due to sepsis and pneumonia are also significantly higher in dialysis patients as compared to the general population [5, 6] with septicemia as the most common cause of death due to infection among dialysis patients [7]. In patients undergoing hemodialysis, hospitalizations for infection have increased 43% since 1993, although the overall hospitalization rate and total hospital days have declined [7]. Patients on dialysis endure infection rates that are more than 26 times higher than that of the general population without Chronic Kidney Disease (CKD) [8]. The repetitive episodes of bloodstream infection (BSI) and sepsis reported in dialysis patients are related to several factors such as old age, diabetes, accumulation of uremic toxins, an impaired immune system, and the high number of invasive procedures they are exposed to [9–12]. In HD, the type of vascular access used is also important [9, 10]. Infection-control programs in dialysis units have, with some success, minimized the risk of infectious complications from bacteria acquired through contamination of dialysis fluids and equipment [13]. Kidney Disease: Improving global outcomes (KDIGO) Anemia work group [14] However, little information on the temporal development of CVD and infective diseases across the last decades is available. Therefore, in this study, we aimed to investigate changes in the occurrence of cardiovascular disease and infective complications in dialysis patients across a 22-year period.

## Methods

### Data

In Denmark, all residents are provided with a permanent unique personal identifier that can be used to link information within and across all nationwide computerized registers, allowing record linkage analysis. The Danish National Patient Registry has since 1977 collected nationwide data on all somatic hospitalizations, hospital wards, procedural codes, primary discharge diagnosis and if appropriate one or more secondary diagnosis.

From 1977 to 1994 the diagnosis code of the disease for each admission has been registered according to the 8th revision of International Classification of Diseases (ICD). From 1994 and onward diagnoses were coded with reference to 10th revision (ICD-10) [15]. The codes assessed for comorbidity are considered valid [16]. Surgical procedures were added in 1996 and are organized by the Nordic Medico-statistical Committee (NOMESCO) [17]. The Danish Civil Registration System holds updated information on all Danish citizens including date of birth, sex and vital status (death and migration) since 1968 [18]. The registration of end stage renal disease (ESKD) patients acquiring renal replacement therapy in Denmark has been available in the Danish National Registry on Regular Dialysis and Transplantation since 1990 [19].

### Population, outcomes and follow-up

All patients with ESKD that initiated peritoneal dialysis and hemodialysis in the period from January 1st, 1996 to December 31st, 2017 were identified from The Danish National Registry on Regular Dialysis and Transplantation.

The day patients initiated dialysis was defined as the index date. From this cohort we identified patients with CVD, pneumonia, infective endocarditis and sepsis. All outcomes were pre-selected. We included their first time admission to a hospital in Denmark in the study period as recorded in the Danish National Patient Registry. We defined the cardiovascular events according to hospital admission with ICD-10 discharge codes for heart failure (HF), ischemic heart disease (IHD), peripheral arterial disease (PAD) and stroke/TCI. The infective events were defined according to hospital admission with ICD-10 discharge codes for pneumonia, sepsis and infective endocarditis (IE). The codes are specified in supplemental Table 1. These codes are considered accurate and valid [15, 16]. The Danish national guidelines on IE follow the European Society of Cardiology guidelines on IE and the modified Duke criteria are used as an helpful algorithm for the diagnosis of IE [20–23].

Subjects were followed until the occurrence of an event, recovery of kidney function, the termination of dialysis treatment, death or end of study (December 31st, 2017) – whichever came first. Recovery of kidney function was defined as regaining of kidney function while receiving chronic HD. Termination of dialysis treatment was defined as end of treatment after initiation of renal replacement therapy without regaining of kidney function. There were no individuals lost to follow-up in the study cohort. All methods were performed in accordance with the relevant guidelines and regulations.

### Statistics

Data are presented as median with 25 and 75 percentiles for continuous variables and as counts and percentages for categorical variables. Continuous variables were compared using Kruskal Wallis test and chi-square was used to compare categorical variables.

The calendar period has been arranged into consecutive five periods: 1) 1996–2000, 2) 2001–2005, 3) 2006–2009, 4) 2010–2013 and 5) 2014–2017 with 1996–2000 being the reference period throughout the analyses. The outcomes – CVD, infective endocarditis, pneumonia and sepsis - were analyzed independently. If a patient in period 1 (1996–2000) did not experience an outcome, the patient continued in period 2 and henceforth. To check for any role of vintage, we conducted a sensitivity analysis for each of the outcomes where each calendar period was analyzed separately and found valid. In this sensitivity analysis only those who initiated RRT within each calendar period were included, and they were censored at the end of the respective calendar periods or death.

For peritoneal dialysis and hemodialysis, respectively, the crude incidence rates were calculated with the nominator being the total number of events occurring in each calendar period and the denominator being the sum of the person-years of the population in each calendar period. In analyses of CVD and infective diseases, each observation was split at any change in renal replacement therapy modality, so that these variables could change time-dependently. The following covariates were included in the separate regression models for HD and PD: sex, age, and calendar time. Rate ratios (RR) and confidence intervals (CI) of 95% were estimated for peritoneal dialysis and hemodialysis using Poisson regressions. Overdispersion of the Poisson regression model was tested by the method suggested by Cameron & Trivvedi [24]. A sub analysis of the distribution of peritonitis as secondary diagnosis among PD patients admitted with sepsis was conducted, and Cochran-Armitage trend test was used to check for any trend. All statistical analyses were performed using R studio version 1.1.447. A *p*-value less than 0.05 was considered significant.

## Results

### Study population

A total of 14,237 patients receiving hemodialysis (HD) and peritoneal dialysis (PD) were identified between 1996 and 2017. The PD-population comprised 4285 patients, of which 63.7% were males. The overall median age at the initiation of PD was 67 years [57–76] and the median age increased across the calendar periods from 65 [57–73] in 1996–2000 to 69 [55–76] in 2014–2017 (Table 1). In the HD-population, including 9952 patients, 69.2% were males. The overall median age was 71 [61–78] without any changes across the calendar periods (Table 2). The Poisson regression model was valid with no signs of overdispersion (Supplemental Table 2).

**Table 1:** Baseline characteristics of patients initiating peritoneal dialysis between the calendar period 1996–2017

	Overall	1996–2000	2001–2005	2006–2009	2010–2013	2014–2017	*P*-Value
Total – no. (%)	4285 (100.0)	790 (100.0)	1031 (100.0)	854 (100.0)	751 (100.0)	859 (100.0)	
Sex – no. (%)							
Male	2729 (63.7)	505 (63.9)	628 (60.9)	526 (61.6)	504 (67.1)	566 (65.9)	< 0.0001
Age, Yr							
Age, [Q1-Q3]	67 [57.0–76.0]	65 [57.0–73.0]	67 [57.0–75.0]	67 [56.0–77.0]	69 [58.0–77.0]	69 [55.5–76.0]	< 0.0001
Age group – no. (%)							
< 50	575 (13.4)	83 (10.5)	133 (12.9)	125 (14.6)	101 (13.4)	133 (15.5)	
50–59	724 (16.9)	163 (20.6)	176 (17.1)	140 (16.4)	101 (13.4)	144 (16.8)	
60–69	1094 (25.5)	246 (31.1)	289 (28.0)	210 (24.6)	176 (23.4)	173 (20.1)	
70–79	1289 (30.1)	255 (32.3)	307 (29.8)	231 (27.0)	233 (31.0)	263 (30.6)	
≥ 80	603 (14.1)	43 (5.4)	126 (12.2)	148 (17.3)	140 (18.6)	146 (17.0)	< 0.0001
Comorbidities – no. (%)							
Cancer	291 (6.8)	50 (6.3)	60 (5.8)	54 (6.3)	50 (6.7)	77 (9.0)	< 0.0001
Diabetes	1249 (29.1)	221 (28.0)	283 (27.4)	240 (28.1)	233 (38.7)	272 (31.7)	0.398
PAD^a^	219 (5.1)	44 (5.6)	69 (6.7)	53 (6.2)	25 (3.3)	28 (3.3)	0.0001
HT^b^	2059 (48.1)	370 (46.8)	537 (52.1)	386 (45.2)	355 (47.3)	411 (47.8)	< 0.0001
AF^c^	287 (6.7)	24 (3.0)	57 (5.5)	57 (6.7)	59 (7.9)	90 (10.5)	< 0.0001
IHD^d^	724 (16.9)	121 (15.3)	193 (18.7)	142 (16.6)	141 (18.8)	127 (14.8)	0.267
CKD^e^	4245 (99.1)	769 (96.6)	1023 (99.2)	847 (99.2)	751 (100)	855 (94.1)	< 0.0001
ICD^f^	44 (1.0)	≤ 3 (< 0.3)	≤ 3 (< 0.3)	9 (1.1)	10 (1.3)	18 (2.1)	0.062
HF^g^	455 (10.6)	83 (10.5)	105 (10.2)	85 (10.0)	79 (10.5)	103 (12.0)	< 0.0001
COLD^h^	199 (4.6)	27 (3.4)	47 (4.6)	34 (4.0)	40 (5.3)	51 (5.9)	0.0005
Prosthetic heart valve	14 (0.3)	≤ 3 (< 0.3)	≤ 3 (< 0.3)	≤ 3 (< 0.3)	4 (0.5)	6 (0.7)	0.195

Ages shown as median and quartiles (median, Q1-Q3), *Yr* Year, *No* Number

^a^ Peripheral Artery Disease, ^b^ Hypertension, ^c^ Atrial Flutter, ^d^ Ischemic Heart Disease, ^e^ Chronic Kidney Disease, ^f^ Cardiac Implantable Electronic Device, ^g^ Heart Failure, ^h^ Chronic Obstructive Lung Disease

**Table 2:** Baseline characteristics of patients initiating hemodialysis between the calendar period 1996–2017

	Overall	1996–2000	2001–2005	2006–2009	2010–2013	2014–2017	*P*-Value
Total – no. (%)	9952 (100.0)	2018 (100.0)	2390 (100.0)	1911 (100.0)	1794 (100.0)	1839 (100.0)	
Sex – no. (%)							
Male	6885 (69.2)	1246 (61.7)	1539 (64.5)	1214 (63.5)	1168 (65.1)	1218 (66.2)	< 0.0001
Age, Yr							
Age, [Q1-Q3]	71 [61.0–78.0]	68 [59.0–76.0]	71 [62.0–78.0]	72 [63.0–80.0]	71 [60.0–79.0]	70 [58.5–77.0]	< 0.0001
Age group – no. (%)							
< 50	1039 (10.4)	224 (11.1)	186 (7.8)	162 (8.5)	213 (11.9)	254 (13.8)	
50–59	1236 (12.4)	319 (15.8)	279 (11.7)	199 (10.4)	207 (11.5)	232 (12.6)	
60–69	2341 (23.6)	541 (26.8)	583 (24.4)	440 (23.0)	386 (21.5)	391 (21.3)	
70–79	3312 (33.3)	672 (33.3)	831 (34.8)	604 (31.6)	563 (31.4)	642 (34.9)	
≥ 80	2024 (20.3)	262 (13.0)	511 (21.4)	506 (26.5)	425 (23.7)	320 (17.4)	< 0.0001
Comorbidities – no. (%)							
Cancer	1315 (13.2)	244 (12.1)	297 (12.4)	253 (13.2)	234 (13.0)	287 (15.6)	< 0.0001
Diabetes	3141 (31.2)	542 (26.9)	733 (30.7)	602 (31.5)	613 (34.2)	651 (35.4)	0.236
PAD^a^	779 (7.8)	155 (7.7)	238 (10.0)	145 (7.6)	131 (7.3)	110 (6.0)	< 0.0001
HT^b^	4521 (45.4)	793 (39.3)	1147 (48.0)	887 (46.4)	827 (46.1)	867 (47.1)	< 0.0001
AF^c^	1105 (11.1)	118 (5.8)	233 (9.7)	211 (11.0)	252 (14.0)	291 (15.8)	< 0.0001
IHD^d^	2061 (20.7)	343 (17.0)	557 (23.3)	446 (23.3)	377 (21.0)	338 (18.4)	< 0.0001
CKD^e^	9529 (95.7)	1936 (95.9)	2336 (97.7)	1826 (95.6)	1707 (95.2)	1724 (93.7)	< 0.0001
ICD^f^	145 (1.5)	≤ 3 (< 0.3)	28 (1.2)	36 (1.9)	38 (2.1)	42 (2.3)	0.227
HF^g^	1680 (16.9)	290 (14.4)	408 (17.1)	307 (16.1)	320 (17.8)	355 (19.3)	< 0.0001
COLD^h^	818 (8.2)	100 (5.0)	203 (8.5)	172 (9.0)	171 (9.5)	172 (9.4)	< 0.0001
Prosthetic heart valve	92 (0.9)	6 (0.3)	18 (0.7)	18 (0.9)	26 (1.4)	24 (1.3)	0.767

Ages shown as median and quartiles, (median, [Q1-Q3]), *Yr *Year, No.Number

^a^ Peripheral Artery Disease, ^b^ Hypertension, ^c^ Atrial Flutter, ^d^ Ischemic Heart Disease, ^e^ Chronic Kidney Disease, ^f^ Cardiac Implantable Electronic Device, ^g^ Heart Failure, ^h^ Chronic Obstructive Lung Disease

### Comorbidities

The distribution of comorbidities in PD and HD is shown in Tables 1 and 2, respectively. At baseline, 48.1% of the overall PD-population had hypertension, 29.1% diabetes mellitus and 16.9% ischemic heart disease. In HD, 45.4% of the patients had hypertension, 31.2% diabetes mellitus and 20.7% ischemic heart disease at baseline. Overall, both PD and HD patients had a higher burden of co-morbidities in the periods 2010–2013 and 2014-2017compared to 1996–2000.

### Changes over time in CVD

**Fig. 1:**
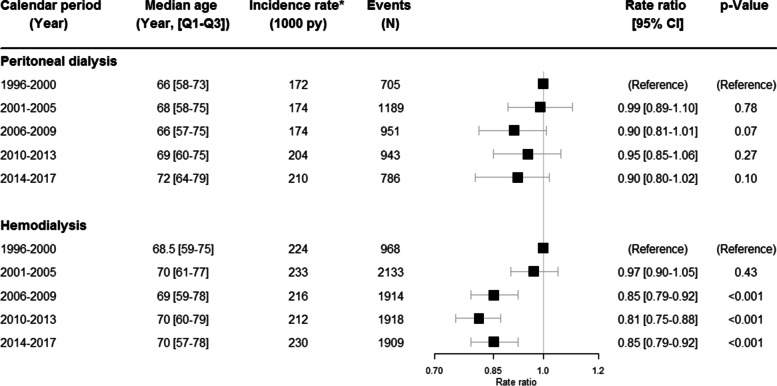
Changes in cardiovascular disease in dialysis over time. The changes over time in cardiovascular disease (CVD) in peritoneal dialysis and hemodialysis. The rate ratios have been adjusted for age and sex.*Unadjusted rates, N = number, py = person-years, CI = confidence interval, Q1-Q3 = 25–75% quartiles

A total of 4574 events of CVD occurred in PD and 8842 events in HD. In the PD population, the rate ratios of CVD did not change significantly, but importantly a decreasing trend up to 10% was seen from start of the observation period in 1996–2000 to the period 2006–2009 (RR 0.90; 95% CI 0.81–1.01, *p* = 0.07) (Fig. 1). Subsequently, the rate ratios of CVD remained constant with similar rate ratios in 2014–2017 (RR 0.90; 95% CI 0.80–1.02), (*p* = 0.102). The crude incidence rates increased from 172 per 1000 person-years in 1996–2000 to 210 patient years per 1000 person-years in 2014–2017.

In hemodialysis the crude incidence rates were stable, but the rate ratios of CVD decreased significantly in the years 2006–2017 (*P* <  0.001), as compared to the reference period 1996–2000 (Fig. 1) after adjusting age, gender and calendar period. The number of events were almost similar between 2006 and 2017.

Male sex was associated with a higher rate ratio of CVD in PD (RR 1.36; 95% CI 1.28–1.45) as well as in HD (RR 1.19; 95% CI 1.14–1.24). The sensitivity analysis (Supplemental Figure 1) including only those patients initiating HD or PD within each calendar period did not affect the association of the outcome over time in both the dialysis modalities.

### Changes over time in infective diseases

#### Pneumonia

**Fig. 2:**
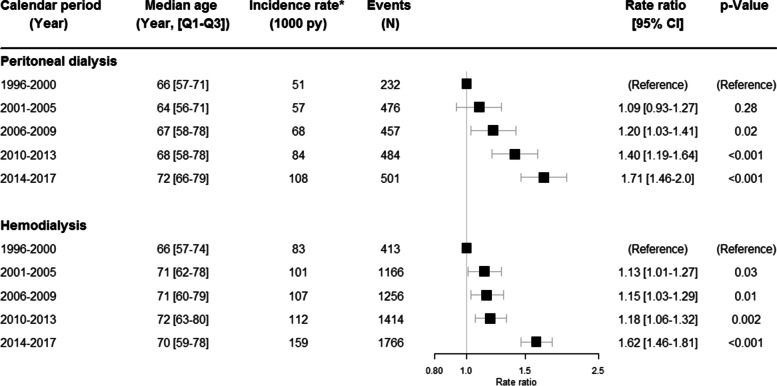
Changes in cardiovascular disease in dialysis over time. The changes over time in pneumonia in peritoneal dialysis and hemodialysis. The rate ratios have been adjusted for age and sex.*Unadjusted rates, N = number, py = person-years, CI = confidence interval, Q1-Q3 = 25–75% quartiles

Two thousand one hundred fifty events of pneumonia were diagnosed in PD and 6015 events in HD across the follow-up period. A progressive increase in the rate ratios of patients in PD was found across the calendar periods, with the highest rate ratio in 2014–2017 (1.71; 95% CI 1.46–2.0) as compared with the reference period (Fig. 2). The changes are significant in the last 3 calendar periods (*p* <  0.001). The crude incidence rates of pneumonia doubled from 51 per 1000 person-years in 1996–2000 to 108 per 1000 patient-years in 2014–2017.

Patients receiving HD had a stable and significant increase in rate ratios of pneumonia between 2001 and 2013 (*p* <  0.05) with a further increase in the rate ratio between 2014 and 2017 (RR 1.62; 95% CI 1.46–1.81, *p* < 0.001) (Fig. 2). The number of events increased throughout all the calendar periods as compared to 1996–2000.

Male sex was associated with a slightly higher rate ratio of pneumonia in HD (RR 1.12; 95% CI 1.06–1.18) but no such association was seen in PD (RR 1.03; 95% CI 0.94–1.13).

#### Infective endocarditis

A total of 97 events of infective endocarditis (IE) were diagnosed in PD patients and 845 events in HD patients. Supplemental Table 3 shows an overview of the distribution of infective outcome diagnosis as secondary discharge diagnosis among the PD and HD population for the same admission as their primary IE or pneumonia diagnosis. In PD a significant decrease in the rate ratios of IE was found in the periods 2001–2005 (RR 0.50; 95% CI 0.26–0.94, *p* = 0,03) and 2006–2009 (RR 0.43; 95% CI 0.21–0.87, *p* = 0.49). In 2010–2013 and in 2014–2017 the rate ratios were almost the same as in the reference period.

The rate ratios of IE in HD were unchanged from 1996 to 2009 (RR 1.14; 95% CI 0.79–1.64). A significant increase in the rate ratios was demonstrated in 2010–2013 (RR 2.02; 95% CI 1.43–2.85) and in 2014–2017 (RR 3.39; 95% CI 2.42–4.75), respectively (*p* < 0.001). The unadjusted incidence rate of IE went from 7 per 1000 person-years in 1991–2000, 2001–2006 and 2007–2009 to 23 per 1000 patient years in 2014–2017.

**Fig. 3:**
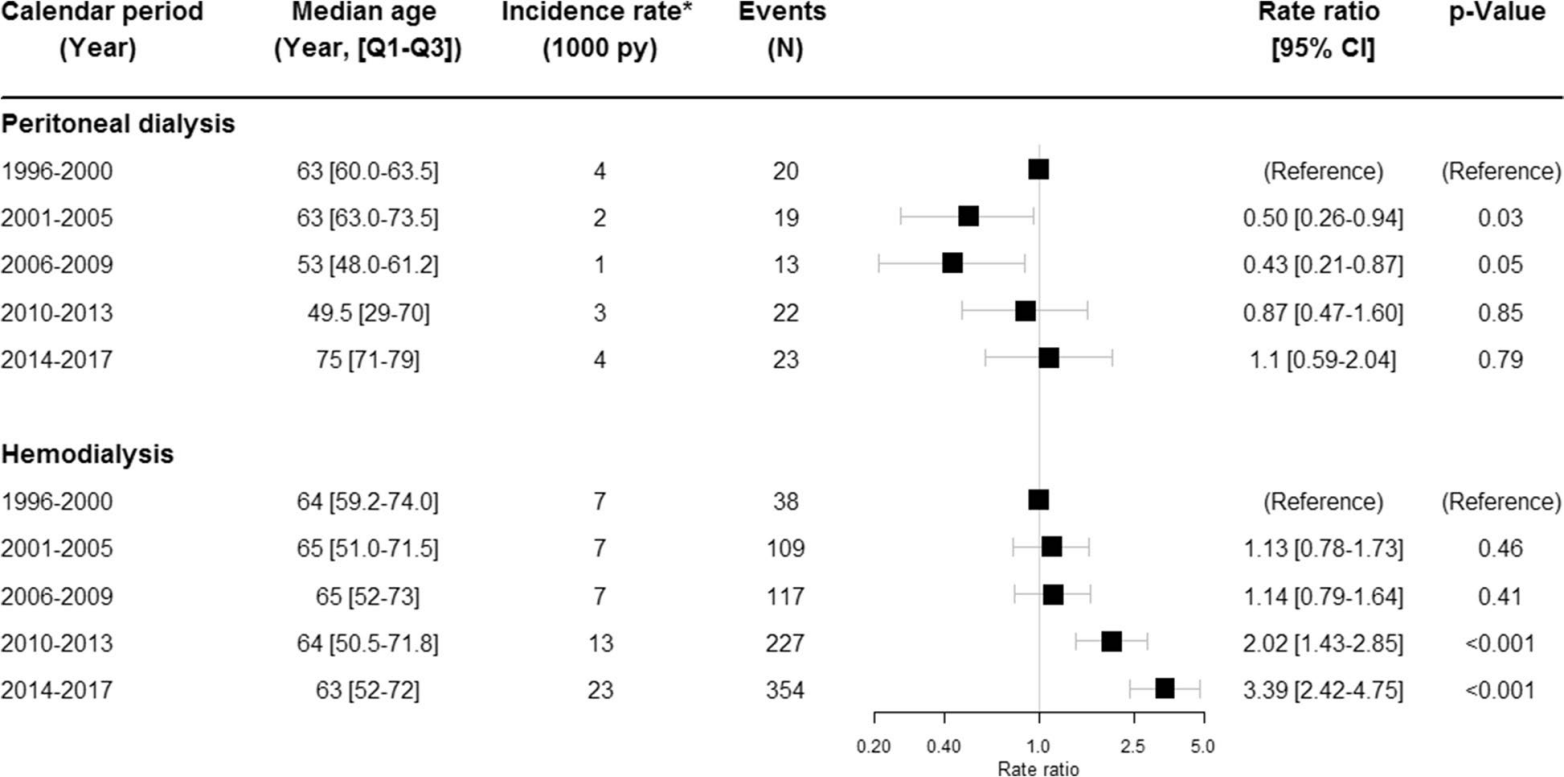
Changes in cardiovascular disease in dialysis over time. The changes over time in infective endocarditis in peritoneal dialysis and hemodialysis. The rate ratios have been adjusted for age and sex.*Unadjusted rates, N = number, py = person-years, CI = confidence interval, Q1-Q3 = 25–75% quartiles

Male sex was not associated with a significantly higher rate ratio of IE, neither in PD (RR 1.30; 95% CI 0.85–2.00) nor in HD (RR 1.05; 95% CI 0.91–1.21). Figure 3 shows the time trend of IE in PD and HD from 1996 to 2017.

#### Sepsis

**Fig. 4:**
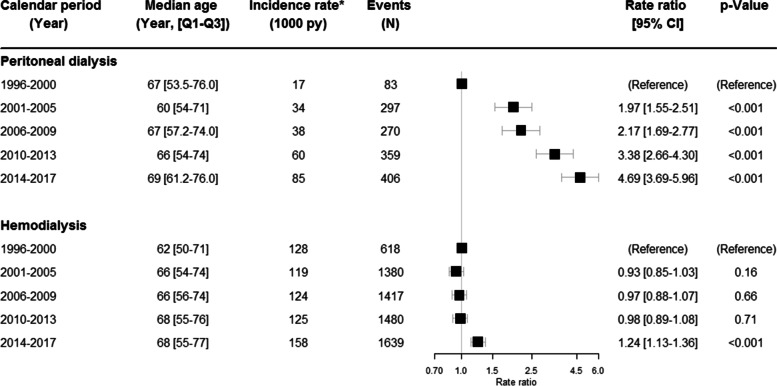
Changes in sepsis in dialysis over time. The changes over time in sepsis in peritoneal dialysis and hemodialysis. The rate ratios have been adjusted for age and sex. *Unadjusted rates, N = number, py = person-years, CI = confidence interval, Q1-Q3 = 25–75% quartiles

A total of 1415 events of sepsis occurred in PD and 6534 events in HD. Figure 4 illustrates the changes in development of sepsis through the years in patients receiving HD and PD.

The PD population had a significant increase in the rate ratios of sepsis throughout the years with an almost 5-fold increase in 2014–2017 (RR 4.69 95% CI 3.69–5.96) as compared to the reference period (*p* < 0.001). The unadjusted incidence rate went from 17 per 1000 person-years in 1996–2000 to 85 per 1000 person-years in 2014–2017.

In the HD group, no significant change in rate ratios were demonstrated in the period 2001–2013 as compared to the reference calendar period. In 2014–2017 a smaller but significant (*p* < 0.001) increase was revealed (RR 1.24; 95% CI 1.13–1.36). The number of events increased throughout the calendar periods.

Male sex was associated with almost the same rate ratio of developing sepsis in HD (RR 1.04; 95% CI 0.99–1.09) as the female population whereas it was slightly higher in PD (RR 1.12; 95% CI 1.01–1.25). A sub analysis of the distribution of peritonitis as secondary diagnosis among PD patients admitted with sepsis as the primary diagnosis, is seen in Table 3. The results reveal a downwards trend in the prevalence of peritonitis among PD patients admitted with sepsis, decreasing from 7% in 1996–2000 to 3% in 2014–2017, *p* < 0.001.

**Table 3:** Distribution of peritonitis as secondary diagnosis on the same admission as the primary diagnosis of sepsis among PD patients

Calendar period (years)	1996–2000	2001–2005	2006–2009	2010–2013	2014–2017	*P-value
Percentage of peritonitis (%)	7%	6%	6%	5%	3%	< 0.001

*PD* Peritoneal dialysis

## Discussion

In this study we investigated the time trends of CVD, pneumonia, infective endocarditis and sepsis in patients on dialysis. The study covered a 22 years period, divided into five consecutive time intervals. Our major findings were: i) a significant decrease in cardiovascular diseases across the last decade in patients on HD as compared to 1996–2000 (control period) and a decreasing trend up to 10% (non-significant) from 1996 to 2000 to 2006–2009 in PD, ii) a significant increase over time in rate ratios of developing pneumonia in both HD and PD, iii) a significant increase in rate ratios of infective endocarditis in HD patients during the last two calendar periods, and a significant increase in sepsis for PD throughout the calendar periods. A sensitivity analysis with each calendar period being analyzed separately for each outcome did not alter the time trends in CVD, pneumonia, IE and sepsis.

In 2003, the Hemodialysis Study (HEMO) in the United States reported that 40% of dialysis patients had cardiovascular disease at study entry and that 63% of admissions to hospitals due to cardiovascular reasons was caused by coronary artery disease [25]. Likewise, in our study the baseline characteristics also showed a dialysis population with a high percentage of CVD.

Only few studies have investigated the temporal trend of CVD in dialysis patients. From an inpatient nationwide database from United States, Alqahtani et al. analyzed patients with ESRD on maintenance dialysis (HD and PD) admitted with the diagnosis of acute ischemic stroke in the period 2003–2014, as compared to the general population [26]. The study found a significant decrease in the incidence of acute ischemic stroke in the patients on maintain dialysis between 2003 and 2014 (P_trend_ < 0.001), while it remained stable in the non-dialysis population (P_trend_ = 0.78).

A large Japanese study by Wakasugi et al. analyzed mortality trends among Japanese dialysis patients in the period 1988–2013 [27]. They found an improved survival rate, primarily due to a decrease in cardiovascular mortality from 106.9 per 1000 person-years in 1988 to 39.9 per 1000 person-years in 2013. In the past 3–4 decades there has been a general increase in our knowledge of how to prevent and treat CVD and its risk factors. Primary preventions with more focus on healthy diet, physical activity, avoidance of tobacco/alcohol as well as modifying other risk factors and implementation of new interventional and medical therapeutic strategies are responsible for a reduction in CVD morbidity and mortality globally [28]. This could also be part of the explanation of a reduction in rate ratios of CVD among dialysis patients, as demonstrated in these studies and in our study.

To our knowledge, there are no larger time trend studies examining the risk of pneumonia or sepsis in patients on dialysis. In a single hospital-based dialysis program Berman et al. reviewed 433 patients with infections and ESRD requiring long-term dialysis between 1992 and 2000, and found pneumonia as the source of 13% of the infections [29]. In another retrospective, observational cohort study, Sibbel et al. analyzed patients receving dialysis between 2009 and 2011 with episodes of pneumonia [30]. They found an overall incidence rate of 21.4 events per 100 patient-years but data on temporal trends were not reported. Stratification by age revealed that incidence rates of pneumonia were higher among older patients. Gup et al. demonstrated in a retrospective study including 289,210 patients that within a year of initiating dialysis therapy, 21% of the study population (60,610 patients) developed pneumonia and had an overall event rate of 27.9 events per 100 patient-years [6]. The rates were 59% highere in HD as compared to PD (*P* < 0.0001). In our study we also found high rates of pneumonia in both the HD and the PD population. Additionaly, we found increasing rate ratios thoughout the time periods for both dialysis modalities, as compared to the reference interval. Since sepsis may be a complication to IE or pneumonia we determined the frequency of sepsis recorded as secondary discharge diagnosis for each IE and pneumonia admission, respectively. However, less than 3% of patients with IE as primary diagnosis had a secondary diagnosis of sepsis. Similarly, 5% or less patients with a primary diagnosis of pneumonia had a secondary diagnosis of sepsis. The overlap for the other infective outcomes was also very limited.

Though the mortality rate in IE is well studied [31–34], there are limited large-scale studies examining the risk of IE over the years in ESRD. Bhatia et al. examined outcomes of 44,816 patients with IE on dialysis and 202,547 patients with IE not on dialysis between 2006 and 2011 [35]. This study found increased incidence rates of hospitalizations for IE in the dialysis population from 175/10,000 ESRD patients in 2006 to 222/10,000 ESRD patients in 2011 (*P* = 0.04) with *staphylococcus aureus* being the most frequent microorganism (61%). Chaudry et al. showed an increased incidence rate of IE in patients with ESRD from 1996 to 2012 when adjusted for the increase in the general population during the same period [36]. The risk of IE in HD was higher (Hazard Ratio (HR) 5.46; 95% CI 3.28–9.10) as compared to patients on PD. Chaudry et al. also showed an increase in the relative time of central venous catheter (CVC) during the cohort period 1996–2012 among IE patients with ESRD, but in our study we did not have access to data on the usage of CVC or CVC line infections to determine whether the trend in IE among our HD patients is associated with an use of CVCs. Previous studies showed that HD patients have a high incidence of bacteremia [8, 37] as they are exposed to different types of infections due to vascular access (central venous catheter, arteriovenous fistula and arteriovenous graft) [38] and higher risk of procedure-related bacterial contamination [9, 11, 39]. Furthermore, an increase in the physicians’ awareness for IE along with the guidelines recommending standardized echocardiography in the presence of especially bacteremia in dialysis patients may contribute to the incline in IE observed in the dialysis population [21, 40].

In the HD patients of our cohort the rate ratios of sepsis were unchanged during most of the observation period, with a 25% increase in the last and most recent time interval. In contrast, rate ratios of sepsis in PD increased progressively across the years with a subanalysis revealing a significant decrease (*p* < 0.001) in prevalence of peritonitis among PD patients admitted with sepsis (7% in 1996–2000 to 3% in 2014–2017). Old age, impaired immune system among a fragile patient group and a high number of comorbidities increase the risk of infections [41]. Powe et al. conducted a longitudinal cohort study of patients initiating dialysis in 1986 or 1987 admitted to hospital for septicemia [11]. The follow-up period was 7 years. A total of 4005 patients were on HD and 913 on PD. During follow-up, 11% of the patients experienced at least one episode of septicemia. In a retrospective study, Sakhuja et al. identified patients on maintenance dialysis with severe sepsis from an inpatient nationwide database in United States during the period 2005–2010. They found unadjusted incidence rates of 145.5 per 1000 patient years in patients on dialysis [42]. The study also identified maintenance dialysis as an independent predictor of death in severe sepsis (OR 1.26; 95% CI 1.23–1.29). Sarnak et al. compared annual mortality rates caused by sepsis in patients with ESRD with those in the general population between 1994 and 1996 [5]. After stratification for age, mortality secondary to sepsis remained approximately 100-fold higher in patients with ESRD treated by dialysis compared to the general population. Foley et al. analyzed ESRD patients admitted with septicemia in the first year of dialysis therapy between 1991 and 1999 [43]. Of these 46.5% had septicemia as the primary hospitalization diagnosis. The rates were lower in the PD population (from 5.7 per 100 patient-years in 1991 to 8.0 per 100 patient years in 1999) as compared to the HD population (from 11.6 per 100 patient-years in 1991 to 17.5 per 100 patient years in 1999). These data cannot be directly compared to our study as we did not compare hemodialysis with peritoneal dialysis and used the calendar period 1996–2000 as the reference period. However, our study also showed the tendency with overall lower crude rates in the PD population (17 per 1000 person-years in 1996–2000 to 85 per 1000 person-years in 2014–2017), while the crude rates were overall higher in the HD population (128 per 1000 person-years to 158 per person-years in 2014–2017) at any given calendar period. Thus, despite improved equipment, best practice for catheter care, usage of prophylactic antimicrobial catheter lock solutions and regularly updated hygiene guidelines for the health professionals in the dialysis unit to minimize infections [13], the trend of infections remains upwards.

### Strengths and limitation

There are limitations inherent to the observational design of the study alongside the lack of inclusion of laboratory data,blood culture isolates and information on dialysis treatment factors such as interdialytic weight gain and ultrafiltration rates. Furthermore, this study did not include kidney transplant patients due to the very low number of events across the time periods (Supplemental Table 4). However, the major strengths of our data are the long follow-up period of 22 years and the large study population. Furthermore, the data were extracted from validated nationwide registries including all patients in Denmark on renal replacement treatment in the study period, with no loss of follow-up. The population-based nationwide design reduces the risk of referral bias. Hence, the findings can be broadly generalizable to the Danish dialysis population. Though the analysis of nationwide registries suggests stable use of diagnosis in Denmark, it cannot be excluded that changes in diagnostic criteria and use of more sensitive diagnostic methods over time could have an impact on the interpretation of secular trends in incidence.

## Conclusion

Across the two last decades rate ratios of CVD have decreased significantly in HD, but remained unchanged in PD, as compared to the reference period 1996–2001. In contrast, the time trend of developing pneumonia and sepsis has been constantly upward in both PD and HD, and rate ratios of IE also increased in HD.

## Supplementary Information

**Additional file 1.**

## Data Availability

The data that support the findings of this study are available from Statistics Denmark and Danish Society of Nephrology, but restrictions apply to the availability of these data, which were used under license for the current study, and so are not publicly available. Data are however available from the authors upon reasonable request and with permission of Statistics Denmark and Danish Society of Nephrology. The author Kamal Preet Kaur should be contacted regarding requests of the data from this study.

## Abbreviations

ESKD: End-stage kidney disease
CKD: Chronic kidney disease
IE: Infective endocarditis
CVD: Cardiovascular disease
PD: Peritoneal dialysis
HD: Hemodialysis
BSI: Bloodstream infection
RR: Rate ratio
CI: Confidence interval
ICD: International Classification of Diseases
NOMESO: Nordic Medico-statistical Committee
