# Supplementary material for: Temporal changes in cardiovascular disease and infections in dialysis across a 22-year period: a nationwide study
Source: BMC Nephrol. 2021 Oct 15;22:340. doi: 10.1186/s12882-021-02537-1 (PMC8518158; doi:10.1186/s12882-021-02537-1)
Supplement: Supplementary file 1 — Additional file 1. [file 12882_2021_2537_MOESM1_ESM.docx]

**Supplemental material**

| **Supplemental Table 1: ICD-, procedure- and ATC-codes used to define comorbidities and outcome in the study population** | | |
| --- | --- | --- |
| **Diagnosis** | **Type*** | **Codes** |
| **Comorbidities:** | | |
| Cancer | ICD-10  ICD-8 | DC00-DC97  109-140 |
| Diabetes | ATC | A10 |
| Hypertension | ICD-10  ICD-8 | DI10-DI15  40009, 40019, 40029, 40039, 40099, 40199, 40299, 40399, 40499 |
| Chronic Obstructive Lung Disease | ICD-10  ICD-8 | DJ42-DJ44  49100-49104, 49108, 49109, 49200,49201, 49208, 49209 |
| Cardiac Implantable Electronic Device | NOMESCO | BFCA0, BFCB0 |
| Prosthetic heart valve | NOMESCO | KFJF, KFKD, KFKH, KFMD, KFMH, KFGE |
| Atrial Flutter | ICD-10  ICD-8 | DI48  42793, 42794 |
| Chronic Kidney Disease | ICD-10          ICD-8 | DN02-DN08, DN11, DN12, DN14, DN18, DN19, DN26, DN158-DN164, DN168, DQ61, DE112, DE132, DE142, DE120, DM321B, DQ612, DQ613, DQ615, DQ619  24902, 25002, 58200-58202, 58208, 58209, 58300-58302, 58308, 58309, 58499, 59009, 59320, 75310, 75311, 75319, 79299, 40399, 40499, 44609, 44629 |
| **Cardiovascular disease:** | | |
| Heart Failure | ICD-10  ICD-8 | DI42, DI50, DJ81  42599, 42709, 42710, 42711, 42719 |
| Ischemic Heart Disease | ICD-10  ICD-8 | DI20-DI25  41009, 41099, 41109, 41199, 41209, 41299, 41309, 41399, 41409, 41499 |
| Peripheral Arterial Disease | ICD-10  ICD-8 | DI70  44009, 44019, 44020, 4021, 44028, 44029, 44030, 44039, 44099 |
| **Infective disease:** | | |
| Endocarditis | ICD-10 | DI33, DI330, DI38, DI389, DI339, DI39, DI398 |
| Sepsis | ICD-10 | DA40, DA41, DA499A, DR572 |
| Pneumonia | ICD-10 | DJ13, DJ14, DJ15, DJ18 |
| *ICD: International Classification of diseases, NOMESCO: The Nordic Medico-Statistical Committee, ATC: Anatomical Therapeutic Chemical | | |

| Supplemental table 2: Test of overdispersion | | | |
| --- | --- | --- | --- |
|  | Dialysis modality | Dispersion value | p-value |
| CVD | PD | 0.971 | <0.001 |
|  | HD | 0.963 | <0.001 |
| Pneumonia | PD | 1.034 | <0.001 |
|  | HD | 1.065 | 0.02 |
| IE | PD | 0.970 | 0.01 |
|  | HD | 0.966 | <0.001 |
| Sepsis | PD | 0.997 | 0.04 |
|  | HD | 0.991 | <0.001 |
| *P-value <0.05 is considered significant.*  *CVD = cardiovascular disease; IE = infective endocarditis; HD = hemodialysis; PD = peritoneal dialysis* | | | |

| Supplemental table 3: Secondary diagnosis among PD and HD on the same admission as their IE or pneumonia as primary diagnosis | | | | |
| --- | --- | --- | --- | --- |
| Secondary diagnosis | Patients with primary diagnosis Infective Endocarditis (IE) | | Patients with primary diagnosis Pneumonia | |
|  | Peritoneal dialysis | Hemodialysis | Peritoneal dialysis | Hemodialysis |
| Pneumonia (%) | < 3% | < 3% | - | - |
| Sepsis (%) | < 3% | < 3 % | 4% | 5% |
| IE (%) | - | - | < 3% | < 3% |
| *IE = infective endocarditis; % = percentage of; PD = peritoneal dialysis; HD = hemodialysis* | | | | |

| Supplemental table 4: Number of events of CVD, pneumonia, infective endocarditis (IE) and sepsis among kidney transplant patients. | | | | | |
| --- | --- | --- | --- | --- | --- |
| Outcome (N) | **Calendar period (years)** | | | | |
|  | 1996-2000  (n = 721) | 2001-2005  (n = 818) | 2006-2009  (n=511) | 2010-2013  (n=693) | 2014-2017  (n=579) |
| CVD | 8 | 11 | 13 | 17 | 6 |
| Pneumonia | < 3 | 11 | 7 | < 3 | <3 |
| IE | < 3 | < 3 | < 3 | < 3 | < 3 |
| Sepsis | < 3 | <3 | 4 | 15 | 22 |
| *N = number of events; CVD = cardiovascular disease, IE = infective endocarditis; n= number of patients initiating a kidney transplant process* | | | | | |

**
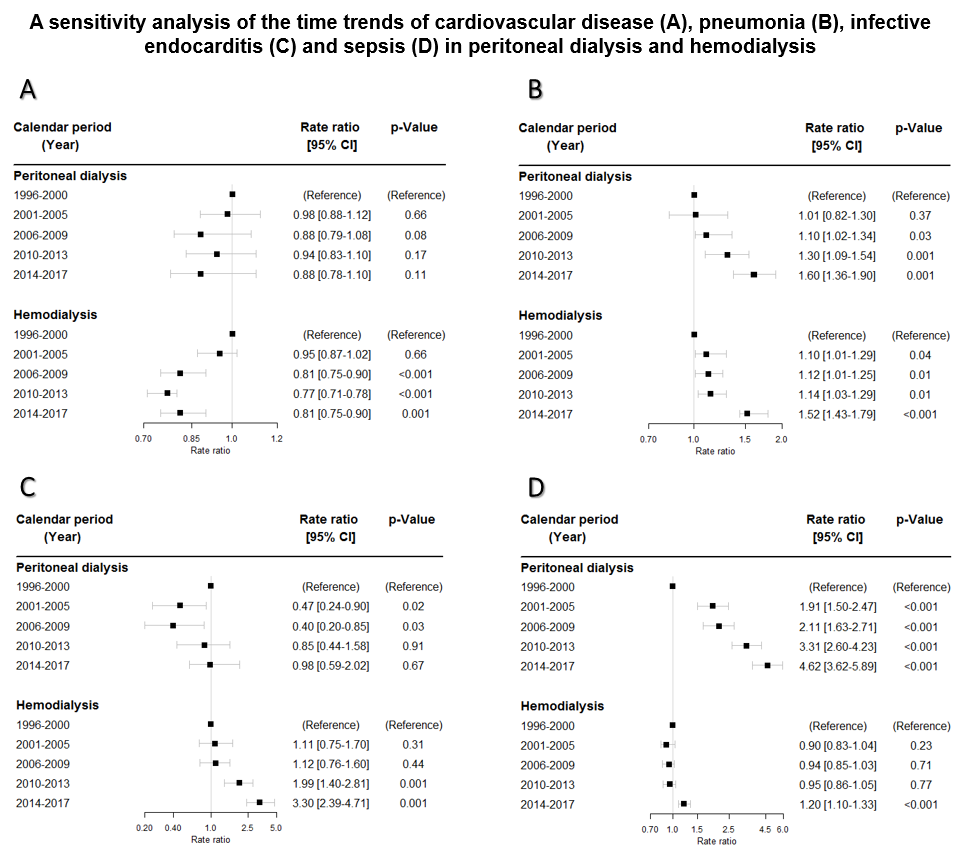
**

***Supplemental Figure 1:*** *Each calendar period has been analyzed separately. Rate ratios have been adjusted for age, gender and calendar period. CI = confidence interval*
